# Blood markers of oxidative stress predict weaning failure from mechanical ventilation

**DOI:** 10.1111/jcmm.12475

**Published:** 2015-04-09

**Authors:** Cléber Verona, Fernanda S Hackenhaar, Cassiano Teixeira, Tássia M Medeiros, Paulo V Alabarse, Tiago B Salomon, Ártur K Shüller, Juçara G Maccari, Robledo Leal Condessa, Roselaine P Oliveira, Silvia R Rios Vieira, Mara S Benfato

**Affiliations:** a Biophysics Department, Program of Cellular and Molecular Biology, Federal University of Rio Grande do SulRio Grande do Sul, Brazil; b Department of Critical Care, Moinhos de Vento HospitalRio Grande do Sul, Brazil; c Federal University of Health Sciences of Porto Alegre – UFCSPA Medical SchoolRio Grande do Sul, Brazil; d Clínicas Hospital of Porto AlegreRio Grande do Sul, Brazil

**Keywords:** Oxidative stress, intensive care units, weaning, mechanical ventilation, malondialdehyde, nitric oxide, vitamin C

## Abstract

Patients undergoing mechanical ventilation (MV) often experience respiratory muscle dysfunction, which complicates the weaning process. There is no simple means to predict or diagnose respiratory muscle dysfunction because diagnosis depends on measurements in muscle diaphragmatic fibre. As oxidative stress is a key mechanism contributing to MV-induced respiratory muscle dysfunction, the aim of this study was to determine if differences in blood measures of oxidative stress in patients who had success and failure in a spontaneous breathing trial (SBT) could be used to predict the outcome of MV. This was a prospective analysis of MV-dependent patients (≥72 hrs; *n* = 34) undergoing a standard weaning protocol. Clinical, laboratory and oxidative stress analyses were performed. Measurements were made on blood samples taken at three time-points: immediately before the trial, 30 min. into the trial in weaning success (WS) patients, or immediately before return to MV in weaning failure (WF) patients, and 6 hrs after the trial. We found that blood measures of oxidative stress distinguished patients who would experience WF from patients who would experience WS. Before SBT, WF patients presented higher oxidative damage in lipids and higher antioxidant levels and decreased nitric oxide concentrations. The observed differences in measures between WF and WS patients persisted throughout and after the weaning trial. In conclusion, WF may be predicted based on higher malondialdehyde, higher vitamin C and lower nitric oxide concentration in plasma.

## Introduction

Reactive oxygen species exert significant influence on contractile function of skeletal muscles of the respiratory system and the limbs 1. Both types of skeletal muscle constitutively generate reactive oxygen species and have comparable levels of antioxidant buffers 2. In animals, diaphragmatic inactivity associated with mechanical ventilation (MV) leads to muscle fibre atrophy in the diaphragm and to a reduction in diaphragmatic force-generating capacity 3–12. Oxidative stress is thought to be a key factor contributing to MV-induced respiratory muscle dysfunction (mainly diaphragmatic), and probably increases MV time and makes the weaning process difficult 1,2,9.

In MV-subjects, Jaber *et al*. 13 showed tissue atrophy and increased expression of ubiquitinated proteins, nuclear factor-κB and calpain isoforms measured in diaphragmatic muscle; similarly, Levine *et al*. 14 found marked muscle atrophy and increased diaphragmatic proteolysis (reduced glutathione concentration and increased active caspase-3). Both groups made measurements in muscle diaphragmatic fibre during MV-support. As it is impractical to obtain biopsies of diaphragm for routine diagnostic purposes, it would be useful to employ correlative blood measures for diagnosis. There are, however, no reports indicating differences in blood measures of patients who failed or were successfully weaned from ventilation. Thus, the aim of this study was to determine if differences in blood measures of oxidative stress in patients who had success or failure in a spontaneous breathing trial (SBT) could, in the future, be used to predict the outcome of MV.

## Material and methods

### Study setting

This study was performed within the 44-bed mixed ICU of the *Clínicas* Hospital, a 795-bed tertiary referral hospital in the city of Porto Alegre, Brazil, and was approved by the Research Ethics Committees. All participants or their surrogates provided written informed consent to participate in the study. The ICU staff physicians were blinded to the study design and to measurements obtained, except for arterial blood gas values.

Mechanical ventilation-dependent patients (≥72 hrs; *n* = 34) undergoing the institutional weaning protocol between March 2009 and October 2010 were included. Exclusion criteria were: (*i*) refusal to sign the consent form, (*ii*) current use of multivitamin or iron supplements, (*iii*) neurodegenerative diseases (Parkinson's disease, Alzheimer syndrome, amyotrophic lateral sclerosis) and (*iv*) tracheotomized patients.

### Weaning protocol

Patients were ventilated on a Servo 900C, Servo 300 (Siemens-Elema AB, Solna, Sweden) or Evita-4. They were assessed daily for the presence of the following readiness-to-wean criteria: (*i*) improvement in the underlying condition that led to acute respiratory failure; (*ii*) adequate oxygenation, indicated by PaO_2_ ≥60 torr (≥8 kPa) on FiO_2_ ≤0.4 and positive end-expiratory pressure ≤8 cm H_2_O; (*iii*) cardiovascular stability (heart rate ≤130 beats/min. and no or minimal vasopressors); (*iv*) afebrile; (*v*) adequate mental status (arousal, Glasgow Coma Scale score of ≥13, and no continuous sedative infusions); (*vi*) effective cough and (*vii*) normal acid base and electrolytes 15.

The weaning protocol was initiated when patients met the readiness-to-wean criteria (described above) and were able to tolerate pressure-support ventilation. Patients were receiving pressure-support ventilation at the time data collection commenced. Baseline recordings and blood samples were obtained first during 10 min. of MV, and then the SBT was initiated. The patient was placed in a semi-recumbent position and breathed through a T-tube circuit, receiving the same FiO_2_ concentration as during MV. Intolerance to SBT was defined as exhibiting one of the following: respiratory rate ≥35 breaths/min., oxygen saturation by pulse oximetry (SpO_2_) ≤88%, heart rate ≥140 beats/min. or changes ≥20%, change in mental status (drowsiness, coma, agitation, anxiety), diaphoresis, accessory muscle activity or thoracoabdominal paradox. Patients exhibiting any of these criteria were returned to MV, and the test was scored as weaning failure (WF). Patients without these features at the end of the trial were extubated, and the test was scored as weaning success (WS). Decisions for extubation or return to MV were made by ICU staff physicians, guided by the institutional weaning protocol.

### General ICU support

Patients in both groups were treated similarly without any kind of differential clinical management. Patients received a commercial diet prescribed by the ICU nutrition support team *via* nasoenteral tube. The amount of calories prescribed was 35–40 kcal/kg/day; the amount of vitamin C provided by the diet was 42 mg/100 ml. Treatment of infections and renal dialysis (when needed) were the same in both groups. No patients received immunotherapy. During the MV-period, the protocol for sedation was the same for both groups; only two sedatives were prescribed (fentanyl and midazolam).

### Data collection

Collection of 10 ml of venous blood took place through a central venous catheter for laboratory and biochemical analyses at three time-points: (*i*) during MV-support, immediately before SBT, (*ii*) at 30 min. of SBT in WS patients or immediately before return to MV in WF patients and (*iii*) 6 hrs after the end of SBT. Blood was collected in three vials, containing: coagulation activator, EDTA and citrate. Samples from the remaining tubes were centrifuged, aliquoted and frozen at −80°C for analysis of oxidative stress. Haemolysates were prepared by lysing red blood cells with 2% ethanol (ratio 1:10) followed by centrifugation to obtain crude extracts. Clinical laboratory analysis of ventilatory, haemodynamic, blood gas and biochemical parameters [serum transferrin saturation, serum ferritin, serum iron, serum total iron-binding capacity and uric acid levels] were requested routinely on ICU patients, and results were transcribed into their medical records. Additional tests (described below) were performed in the research laboratory.

### Biochemical markers of injury

Oxidative damage in proteins was measured in plasma by determining the carbonyl groups by a previously validated method 16. Plasma carbonyl was normalized to total protein. The assessment of damage in lipids was analysed in plasma by measuring malondialdehyde (MDA), a product of lipid peroxidation. Quantification of MDA was carried out by high-performance liquid chromatography (HPLC) 17.

### Evaluation of enzymatic defences

The defence enzymes in erythrocytes measured in this study were: catalase (CAT), superoxide dismutase (SOD) and glutathione peroxidase (GPx). CAT activity was determined on a spectrophotometer by monitoring the disappearance of H_2_O_2_ at 240 nm by the Aebi method 18. SOD activity was measured using a RanSOD® kit (Randox, Crumlin, UK). Enzymatic kinetics of GPX were assessed by Ransel® Kit (Randox). The results are given in units/g haemoglobin.

### Evaluation of non-enzymatic defences

The non-enzymatic defences measured were: total erythrocyte glutathione (tGSH), oxidized glutathione (GSSG) and reduced glutathione (GSH). Vitamin C levels were quantified in plasma, and nitrites and nitrates were determined in both erythrocytes and plasma. The tGSH concentration was analysed using a glutathione assay® kit (Cayman Chemicals, Ann Arbor, MI, USA). Concentrations of reduced glutathione were determined by an alternate protocol following derivatization of GSH with vinylpyridine using the same Kit. Quantization of nitrates and nitrites was performed by a spectrophotometric assay using the method of Grisham 19. The results are reported per gram haemoglobin. Vitamin C was measured by HPLC as described 17.

### Statistical analysis

Variables with normal distribution are presented as mean ± SD, and compared by Student's *t*-test. Variables with non-normal distribution are presented as median (25–75%), and compared by Mann–Whitney test. A comparison of the variables over time in patients who achieved success or failure was performed by the method of generalized estimating equations. All analyses were performed at a 0.05 level of significance. A software package was used for all calculations (SPSS version 18.0.0, SPSS, Chicago, IL, USA).

## Results

Baseline characteristics of patients are showed in Table1. Weaning failure occurred in 38% of patients. The average time to failure was 23 ± 16 min.

**Table 1:** Baseline characteristics

Characteristics	Weaning success (*n* = 21)	Weaning failure (*n* = 13)	*P*
Age, years	63 ± 15	66 ± 16	
Male sex – *n* (%)	12 (57)	6 (46)	
APACHE II score*	23 ± 8	29 ± 12	0.015
Cause of acute respiratory failure – *n* (%)			
Cardiogenic pulmonary oedema	1 (4.8)	2 (15.4)	
Stroke	8 (38.1)	2 (15.4)	
Post-operative	3 (14.3)	3 (23.0)	
ARDS	7 (33.3)	6 (46.0)	
Cardiorespiratory arrest	2 (9.5)	__	
Comorbidities – *n* (%)			
AIDS	2 (9.5)	__	
Diabetes	3 (14.3)	3 (23.1)	
End-stage chronic renal failure	4 (19.0)	2 (15.4)	
Hypertension	9 (42.9)	9 (69.2)	
Ischaemic heart disease	3 (14.3)	2 (15.4)	
Cirrhosis	3 (14.3)	1 (7.7)	
Cancer	1 (14.8)	__	
Arterial gas parameters			
pH	7.46 ± 0.064	7.46 ± 0.074	
pO_2_, mmHg	130 ± 32	127 ± 49	
pCO_2_, mmHg	36.6 ± 6.0	36.3 ± 8.4	
Days on MV before weaning trial	6.8 ± 3.7	8.6 ± 8.5	
Failure time with trial T, min.	__	23 ± 16	
Biochemical results			
Haemoglobin, g/dl	9.23 ± 2.13	8.86 ± 1.87	
Leucocyte count, ×10^3^/μl	11.84 ± 6.43	11.16 ± 4.78	
Glucose, mg/dl	116 ± 26.90	130.85 ± 37.71	
Triglycerides, mg/dl*	157.95 ± 86.17	111.08 ± 44.19	0.045
Total cholesterol, mg/dl	129.19 ± 44.43	127.08 ± 40.92	
HDL cholesterol, mg/dl	23.38 ± 10.17	30.15 ± 18.85	
C-reactive protein, mg/dl	92.26 ± 70.31	109.47 ± 69.42	
Uric acid, mg/dl	3.14 ± 1.37	4.00 ± 1.76	
Transferrin saturation, %	28.74 ± 15.70	27.14 ± 17.91	
Ferritin, ng/ml	1141.68 ± 1337.86	1193.58 ± 2095.20	
Iron, μg/dl	49.81 ± 30.68	45.23 ± 30.07	
Total iron-binding capacity, mg/dl	180.29 ± 59.70	172.15 ± 63.63	

Data are given as mean ± SD unless otherwise noted.

APACHE, Acute Physiology and Chronic Health Evaluation; ARDS, Acute respiratory distress syndrome; AIDS, acquired immunodeficiency disease syndrome; MV, mechanical ventilation.

* *P* ≤ 0.05.

**Figure 1:**
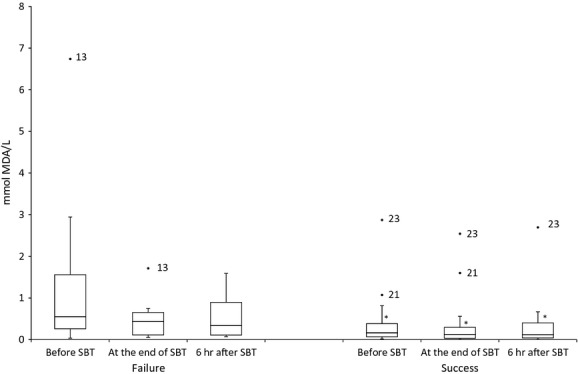
Boxplot showing the changes of serum MDA, before spontaneous breathing trial (SBT), at the end of SBT and 6 hrs after SBT. *P ≤ 0.05 comparing weaning success and failure.

**Figure 2:**
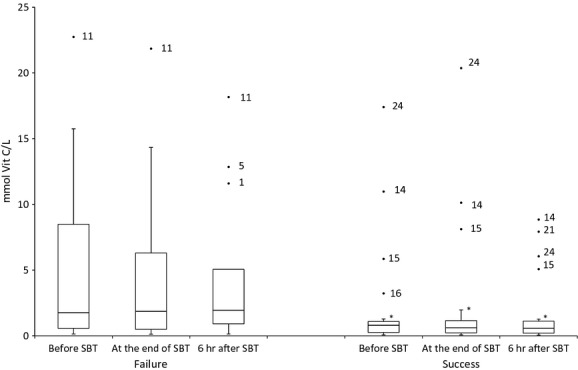
Boxplot showing changes in serum vitamin C before spontaneous breathing trial (SBT), at the end of SBT and 6 hrs after SBT. *P ≤ 0.05 comparing weaning success and failure.

**Figure 3:**
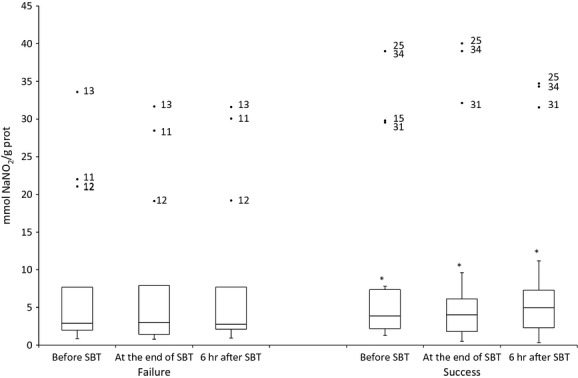
Boxplot showing the changes in plasma nitrite and nitrate before spontaneous breathing trial (SBT), at the end of SBT and 6 hrs after SBT. *P ≤ 0.05 comparing weaning success and failure.

Laboratory findings obtained on samples procured prior to SBT showed that WF patients exhibited damage in lipids [MDA in WF: 0.39 μmol/l (0.14–0.80) *versus* MDA in WS: 0.16 μmol/l (0.06–0.39); Fig.1]; higher antioxidant levels [vitamin C in WF: 1.78 μmol/l (0.52–10.85) *versus* vitamin C in WS: 0.81 μmol/l (0.26–1.20); Fig.2]; and decreased nitric oxide concentrations [nitrite in WF: 1.66 mmol NaNO_2_/g protein (0.87–6.46) *versus* nitrite in WS: 2.29 mmol NaNO_2_/g protein (1.11–4.27); Fig.3]. These differences between WS and WF patients were evident in both the mid- and post-SBT samples.

Evaluations of enzymatic (SOD, CAT and GPx), and other non-enzymatic defences (erythrocyte tGSH, GSSG, GSH, erythrocyte nitrite and nitrate and plasma uric acid), as well as of damage in plasma proteins (carbonyl groups) and iron status were similar in WS and WF patients (Table2).

**Table 2:** Blood biomarkers

	Weaning success (*n* = 21)			Weaning failure (*n* = 13)		
	Before SBT	End SBT	6 h after SBT	Before SBT	End SBT	6 h after SBT
Damage						
Plasma Carbonyl, nmol carbonyl/g prot	0.23 (0.15|0.33)	0.19 (0.15|0.24)	0.20 (0.12|0.24)	0.25 (0.15|0.41)	0.23 (0.15|0.37)	0.18 (0.12|0.28)
Serum MDA, μmol/l*	0.16 (0.06| 0.39)	0.13 (0.03|0.35)	0.11 (0.03|0.37)	0.39 (0.14|0.80)	0.43 (0.10|0.72)	0.37 (0.12|0.90)
Enzymatic defences						
Erythrocytes CAT, U/g Hb	84,612 (55,977|10,071)	82,911 (64,086|108,658)	79,063 (66,444|98,232)	74,865 (58,543|106,417)	87,109 (60,940|114,355)	78,922 (57,487|101,927)
Erythrocytes SOD, U/g Hb	5.11 (4.50|6.45)	5.38 (4.34|6.20)	5.45 (4.37|6.44)	5.80 (4.72|6.35)	5.53 (4.83|6.10)	5.62 (4.76|6.05)
Erythrocytes GPx, U/g Hb	7.49 (4.81|12.0)	7.23 (5.11|13.66)	7.49 (5.17|11.65)	8.36 (6.74|12.3)	9.95 (7.60|11.42)	8.59 (6.48|12.9)
Non- enzymatic defences						
Erythrocytes GSH, μmol/g Hb	5.25 (3.08|9.03)	6.32 (4.45|8.84)	6.34 (4.03|8.31)	5.46 (4.07|8.38)	5.45 (3.29|8.09)	6.38 (4.23|7.73)
Erythrocytes GSSG, μmol/g Hb	7.08 (5.85|8.34)	7.37 (6.49|8.93)	6.78 (6.19|9.07)	7.90 (6.90|8.80)	7.68 (6.23|9.61)	7.64 (6.14|9.18)
Erythrocytes GSH t, μmol/g Hb	13.44 (10.57|14.81)	15.16 (11.24|16.74)	14.88 (9.94|16.31)	13.95 (11.30|15.67)	13.96 (12.28|16.26)	14.21 (10.95|15.78)
Erythrocytes GSH/GSSG	0.778	0.837	0.819	0.755	0.716	0.757
Serum Vitamin C, μmol/l**	0.81 (0.26|1.20)	0.61 (0.22|1.50)	0.60 (0.20|1.20)	1.78 (0.52|10.85)	1.88 (0.49|10.04)	1.95 (0.65|8.34)
Plasma Nit, mmol NaNO_2_/g prot***	2.29 (1.11|4.27)	2.06 (1.05|3.99)	2.69 (1.14|5.08)	1.66 (0.87|6.46)	1.51 (0.77|6.42)	1.86 (1.07|6.49)
Erythrocytes Nit, mmol NaNO_2_/g Hb	80.80 (65.72|93.69)	84.68 (66.23|100.28)	82.19 (70.65|95.81)	80.52 (61.65|96.29)	81.78 (72.67|90.56)	83.24 (65.17|98.46)

Data are given as median (percentiles 25|75).

* *P* = 0.041.

** *P* = 0.045.

*** *P* = 0.022.

The percentage of outliers was taken in account: MDA success/failure (9.5%/7.7%); vitamin C success/failure (23.8%/23.1%); and NO success/failure (19%/23.1%).

The cut-offs to predict success in SBT, and the values of sensibility, specificity, positive and negative predictors are shown in Table3.

**Table 3:** Cut-offs to predict success in SBT

	Value	Sensibility (%)	Specificity (%)	PV+ (%)	PV− (%)
Nit/Nit	>2.0609	64.10	60.30	50.00	73.10
Vit. C	>1.3720	59.00	79.40	63.90	75.80
MDA	>0.3028	58.30	71.00	53.80	74.60

Cut-offs to predict success in SBT, and the values of sensibility, specificity, positive and negative predictors (PV+ and PV− respectively).

## Discussion

The results presented here suggest that oxidative stress is a key mechanism contributing to MV-induced respiratory muscle dysfunction, and probably makes the weaning process difficult. Weaning from MV involves the entire process of liberating the patient from mechanical support and from the endotracheal tube. The management of these patients is difficult, in part, because the pathophysiologic mechanisms responsible for WF are poorly understood 20. Jubran and Tobin 21 showed that inspiratory muscle effort is markedly increased in patients who fail a weaning trial, and the associated increase in intrathoracic pressure excursions may lead to complex cardiopulmonary interactions as demonstrated by Lemaire *et al*. 22, who documented sudden increases in pulmonary artery occlusion pressure during unsuccessful weaning attempts.

A major contributor to WF seems to be the development of respiratory muscle fatigue 23. Most studies suggest that the major factors underlying neuromuscular fatigue occur within the muscle fibres and mainly result from depletion of muscle energy stores or pH changes from lactate accumulation 24,25. Recently, oxygen-derived free radicals have been implicated as mediators of diaphragm muscle dysfunction; however, the precise source of free radicals, the particular physiological conditions under which they can be generated, and the protective mechanism of different free radical scavengers in respiratory muscles remain unclear 26,27.

Another problem for weaning is the early development of ventilator-induced diaphragmatic dysfunction (VIDD) in patients undergoing MV 1,13,14,28. Previous studies 13,14,29, performed on the diaphragms of critically ill patients receiving MV, have found increases in oxidative stress, muscle fibre atrophy and injury, as well as activation of several major proteolytic pathways (ubiquitin–proteasome, caspases, calpains) and up-regulation of the lysosomal-mediated autophagy pathway in the diaphragm. In the present study, we observed differences in the pre-SBT levels of nitric oxide (lower), MDA (higher) and vitamin C (higher) in WF patients compared to WS patients, which suggests that development of VIDD predisposes patients to fail the weaning test. Thus, the SBT merely demonstrated and confirmed pre-existing weakness of respiratory muscles, which is probably associated with the time of dependence on MV.

All patients had very low levels of vitamin C, and this finding is consistent with recognized levels of plasma trace elements and vitamins in critically ill patients 30. Previous data from our research group, using the same measurement technique, showed that outpatients had vitamin C levels three times higher than those shown in this study 31; thus, the presented results were not because of methodological issues. Nutritional support for critically ill patients is often suboptimal, because of problems with both nutrient prescription and delivery. Studies have not yet defined the calorie target for critically ill patients. Patients also receive lesser amounts of food because of diet breaks for the implementation of procedures and routine exams. Further, there are clinical situations where the patient is unable to digest or absorb administered food 32. Added to this, the bioavailability of ascorbic acid is decreased in patients receiving either enteral or parenteral vitamin C 30. Importantly, all patients in this study received the same therapeutic target enteral nutrition, including vitamin C doses of 42 mg/100 ml in the diet 33, and the percentage of patients on renal replacement therapy was the same in both groups. Even after controlling for patients with normal or altered renal function, no statistical differences were observed (data not reported). As vitamin C participates in the degradation of hypoxia-inducible transcription factor in situations of intracellular normoxia 34, we matched vitamin C data with iron metabolism data, but no differences were found.

Although concentrations of vitamin C among all studied patients were lower than found in normal participants, WF patients had vitamin C plasma levels higher than WS patients. We suggest that WF patients were less able to obtain vitamin C from plasma because of down-regulation of vitamin C transporters (Sodium-dependent Vitamin C Transporter) in the diaphragm, as has been reported in cardiomyocytes from rats subjected to injury 35. The lack of transport of vitamin C from plasma into muscle could result in weakness of the diaphragm.

Nitric oxide has multiple physiological roles. For example, nitric oxide synthesized by vascular endothelial cells that line the interior of blood vessels presumably diffuses in all directions, and some of it will reach the underlying smooth muscle. As a result, the muscle relaxes, dilating the vessel and lowering the blood pressure 2. The most common method of measuring of nitric oxide levels is to examine end-products, nitrites and nitrates. It has been suggested that plasma levels of nitrites and nitrates in humans are a measure of vascular endothelial nitric oxide synthesis. In the skeletal muscle of rats, inhibition of nitric oxide production by systemic application of L-NG-Nitroarginine Methyl Ester induced partial hypoxia under resting conditions 36. Therefore, it seems plausible that the robust ‘remaining vasodilatation’, which was found to be insensitive to additional inhibitors, is because of muscle hypoxia rather than to exercise-induced release of activity signals 37. We believe that this is the explanation for why pre-SBT levels of nitrites and nitrates were higher in WS patients than WF patients and did not change during the trial in either group. Nitrites and nitrates can be absorbed from the diet 38. As the patients in this study received a commercial enteral diet, we believe that the measurement of nitric oxide by this technique is reliable.

Animals receiving MV-support exhibit increased lipid peroxidation 8,12,39, which may be decreased by antioxidant administration 40. The WF patients exhibited more lipid peroxidation than WS patients in three measurements. Triglyceride levels were also higher in WF patients, which could lead to a bias in interpreting the MDA results. However, there was an inverse relationship between these findings; patients with higher levels of triglycerides had lower levels of lipid peroxidation. We do not know whether this finding represents specific damage in diaphragmatic muscle cells, as our data were collected from plasma. However, the main finding was that there was a positive relationship between lipid damage and failure to wean from MV.

Our study had a number of limitations. First, the sample size could be too small to detect the real difference between the groups. Second, this is an observational study and, therefore, the results cannot be used to modify clinical practice. However, there are no prior reports using blood measurements of oxidative stress to predicting WS from MV. Analysis of plasma provides for a systemic, rather than diaphragmatic, evaluation of oxidative status, which we believe is appropriate. Because of the small sample size, we were unable to determine a clinically relevant cut-point for the oxidative stress markers that could predict whether the patient would succeed or fail the weaning.

## Conclusions

In the cohort of MV patients studied, higher lipid oxidative damage (MDA), higher vitamin C and lower nitric oxide levels in plasma determined prior to SBT were related to WF from MV.
